# The Shared Pathoetiological Effects of Particulate Air Pollution and the Social Environment on Fetal-Placental Development

**DOI:** 10.1155/2014/901017

**Published:** 2014-11-26

**Authors:** Anders C. Erickson, Laura Arbour

**Affiliations:** ^1^Division of Medical Sciences, University of Victoria, Medical Science Building, Room 104, P.O. Box 1700 STN CSC, Victoria, BC, Canada V8W 2Y2; ^2^Department of Medical Genetics, University of British Columbia, C201, 4500 Oak Street Vancouver, BC, Canada V6H 3N1

## Abstract

Exposure to particulate air pollution and socioeconomic risk factors are shown to be independently associated with adverse pregnancy outcomes; however, their confounding relationship is an epidemiological challenge that requires understanding of their shared etiologic pathways affecting fetal-placental development. The purpose of this paper is to explore the etiological mechanisms associated with exposure to particulate air pollution in contributing to adverse pregnancy outcomes and how these mechanisms intersect with those related to socioeconomic status. Here we review the role of oxidative stress, inflammation and endocrine modification in the pathoetiology of deficient deep placentation and detail how the physical and social environments can act alone and collectively to mediate the established pathology linked to a spectrum of adverse pregnancy outcomes. We review the experimental and epidemiological literature showing that diet/nutrition, smoking, and psychosocial stress share similar pathways with that of particulate air pollution exposure to potentially exasperate the negative effects of either insult alone. Therefore, socially patterned risk factors often treated as nuisance parameters should be explored as potential effect modifiers that may operate at multiple levels of social geography. The degree to which deleterious exposures can be ameliorated or exacerbated via community-level social and environmental characteristics needs further exploration.

## 1. Introduction

Over the last decade, chronic exposure to ambient air pollution has become increasingly recognized as an important risk factor underlying adverse pregnancy outcomes (APOs) [1–9]. In parallel, the associations between socioeconomic status (SES) and APOs are among the most robust findings in perinatal research [10–12], which persist even in settings with universal access to health care [13–16]. While interest in the intersection between health and the social environment is long standing [17–19], renewed attention has been propelled by two independent progressions in quantitative research. The first is the popularization of multilevel statistical models and the ability to separate the individual-level effects from those of their encompassing social and physical environments [20–26]. The second is the emerging research on the biological effects of psychosocial stress on health and its modification by environmental factors. There is now mounting evidence that stress can interact with chemical exposures to exacerbate the toxic effect and the physiological response to a greater extent than either insult (stress or chemical) acting alone [27–31]. Furthermore, the accumulation of low-level exposures to multiple chemicals via multiple sources and pathways shows evidence of dose addition and synergism [32–34]. For example, synergism was observed between aqueous cigarette tar and other respirable particles (e.g., asbestos fibers, particulate matter, and diesel exhaust) [35]. Recognition of these interactions has been incorporated into several conceptual models and study designs of cumulative risk of chemical and nonchemical exposures [36–39] with models recently developed to identify these potentially double-exposed populations [40, 41]. Two complimentary reviews of these models have been recently published [42, 43].

Although the causes of APOs are multifactorial, the placenta plays the main intermediary role between the mother's physical and social environment and the fetus [44–50]. Importantly, a perturbed intrauterine environment inhibiting the fetal growth trajectory may also have long-term adult health implications as suggested by the developmental origins of disease hypothesis [51–53]. Therefore efforts to understand the underlying mechanisms of the physical and social environment that contribute to the disproportionate risk of APOs across the socioeconomic spectrum are required in order to target preventative and restorative interventions. This review will examine how the shared pathoetiological effects of exposure to particulate air pollution and SES act on the fetal-placental unit leading to adverse pregnancy outcomes. This will be accomplished by building on conceptual pathway models of air pollution and SES etiologic mechanisms on APOs [54, 55]. We review the role of the placenta in this context, describing its physiology and obstetrical pathologies followed by a description of particulate air pollution and its toxicokinetics in relation to placentation and how it can lead to APOs. We highlight specific indicators of SES and their biological pathways that intersect with air pollution exposure and how this may contribute to increased susceptibility for APOs. Potential implications and interventions are summarized in the conclusion. Our aim is for this review to be a resource for researchers interested in environmental-perinatal epidemiology. Understanding how correlated social and environmental exposures at times overlap to produce potentially synergistic and modifiable effects will help guide future research and intervention strategies with the aim to improve the overall health of the population [36–40].

## 2. Person, Place, and Context: The Placental, Physical, and Social Environments

### 2.1. The Placenta

The mammalian placenta is an intriguing and remarkable organ. Formed from two genetically distinct organisms, it is multifunctional and vital to fetal development yet situated outside the fetal body with a limited life span. Notable characteristics unique to humans and the Great Apes include deep interstitial implantation and a highly invasive hemochorial phenotype thus allowing the direct interaction of maternal blood and fetal chorionic tissues [56]. Interestingly, this particular aspect of placental evolution has less to do with nutrient transfer efficiency than previously thought and more likely implicates the highly regulated maternal-fetal immunological relationship [57–59].

The first trimester is a critical period in pregnancy involving implantation and initial placentation, two events highly susceptible to disturbance. The “Great Obstetrical Syndromes” [60] such as early/recurrent miscarriage, pregnancy induced hypertension and preeclampsia (PIH/PE), fetal growth restriction (FGR), placental abruption, prelabour rupture of the fetal membranes (PROM), and spontaneous preterm labour may share common etiological mechanisms arising from defective deep placentation (DDP) [61, 62]. Together, these conditions may complicate between 17 and 29% of all pregnancies [63] and are for the purpose of this review referred to collectively as APOs. Furthermore, these conditions may lead to epigenetic programming of adult disease susceptibility including obesity, diabetes, cardiovascular and reproductive diseases, all with their own substantial societal costs [52, 64–66]. DDP refers to the shallow invasion of the placental bed into the maternal decidua and myometrium including incomplete remodeling of the uterine spiral arteries [62, 67]. The latter is a vital event during which under normal conditions the endothelial lining of the spiral artery walls is remodeled to accommodate the inundation of maternal blood flow starting in the second trimester [68]. Spiral arteries that fail to undergo this vascular remodeling are not only narrower in diameter but also remain responsive to vasoconstrictive compounds such as stress hormones. The etiological trigger(s) leading to DDP are thought to involve either early placental oxidative stress which triggers an inflammatory response or, vice versa, an atypical inflammatory maternal immune response to the semi-allogenic fetal-placental unit leading to placental oxidative stress and further inflammation [69, 70]. The difference between a normal and an affected pregnancy is a matter of degrees on a continuum with individual biological and behavioural variability nested within the social and physical environment [12, 24–26, 68, 69, 71–73].

### 2.2. The Physical Environment: Particulate Air Pollution

Air pollution is a general term used to describe the presence of agents (particulates, biologicals, and chemicals) in outdoor or indoor air that negatively impact human health. Several common air pollutants have been associated with APOs, including carbon monoxide (CO), nitrogen dioxide (NO_2_), sulfur dioxide (SO_2_), ozone, particulate matter (PM), and polycyclic aromatic hydrocarbons (PAHs) [1]; however, attention has focused on the latter two compounds showing strong molecular evidence of cytotoxicity, mutagenicity, DNA damage, oxidative stress, and inflammation [55, 74–79]. While the observed risks of APOs in relation to air pollution tend to be modest, the population attributable risk can be quite large due to the pervasiveness of exposure to the general population [9]. Significant risks have been observed even in settings with relatively low ambient air pollution exposure [80, 81]. Therefore, a small increase in risk can have a large public health impact. Preterm birth (PTB) and FGR are major risk factors of perinatal mortality and serious infant morbidities contributing to increased health care and societal costs [82–87].

Particulate matter (PM) is a complex mixture of varying chemical and physical properties. It is defined according to particle size into the inhalable coarse fraction (PM_10_, 2.5–10 *μ*m), the fine respirable fraction (PM_2.5_, ≤2.5 *μ*m), and the ultrafine fraction (UFP, ≤0.1 *μ*m). Their ubiquity and recognized human health risks have deemed them as toxic [88, 89]. Characterizing PM by particle size is important for several reasons. First, particle size dictates the location of deposition in the respiratory system [88, 90]. Second, particle size can give some indication of its general source and behaviour. For example, PM_10_ is mainly derived from mechanical processes such as windblown soil, pollen, minerals and dust from roads, farms, and industrial operations. PM_10_ tends to gravitationally settle in a matter of hours to days. Conversely, PM_2.5_ is a primary by-product of combustion and atmospheric reactions with precursor gases such as SO_2_, nitrogen oxides, ammonia, and volatile organic compounds (VOCs). PM_2.5_ can remain suspended in air for days to weeks and are consequently more prone to long-range transport. Precipitation accounts for 80–90% of PM_2.5_ removal from the atmosphere [88]. Third, the chemical composition is markedly different between PM_10_ and PM_2.5_ mixtures. Derived mainly from the Earth's crust, PM_10_ typically contains oxides of iron, calcium, silicon, and aluminum, whereas PM_2.5_ mixtures derived from anthropogenic combustion sources are mainly composed of sulphates, nitrates, ammonium, trace metals, elemental carbon, and organic hydrocarbons (e.g., PAHs) [88]. Chemical differences and relative proportions also differ within the PM_10_ and PM_2.5_ mixtures with regional (urban-to-rural) and interurban (urban-to-urban) differences as well as intraurban spatial variation [88, 91–93]. Therefore trimester and demographic differences in residential mobility and intraurban population differences are important study design issues to consider [94, 95]. Finally, PM_10_, PM_2.5_, and UFPs differ by their toxicological mechanisms such as their oxidative potential, which may reflect their differences in size, surface area, and/or their chemical constituent compositions, although they tend to be correlated [76, 92, 96, 97]. Transition metals such as copper, nickel, lead, chromium, iron, vanadium, and cobalt among other metals are variably present in ambient air absorbed to PM_2.5_ [92, 93]. Their direct oxidative action or redox potential to create reactive oxidative species (ROS) is one possible mechanism as to how PM_2.5_ induces oxidative DNA and protein damage [78, 97].

There is accumulating evidence that suggests UFPs may be the fraction of PM responsible for many of the adverse health effects reported in air pollution studies [78, 79, 97, 98]. UFPs are a small proportion by mass but make up a large proportion in particle number and have gone either unmeasured or misclassified as PM_2.5_ [88, 98]. Their small size facilitates better tissue penetration deep into lung alveoli and into epithelial cells restricting their clearance via macrophage phagocytosis [98]. Animal studies have shown that UFPs can translocate across the lung epithelium into blood circulation and accumulate in other organs, including the liver, spleen, kidneys, heart, brain, and reproductive organs [98]. The high surface area of UFPs favours the absorption of PAHs and possibly transition metals which has shown to localize in the mitochondria inducing major structural damage. This could be a possible explanation to UFP's exhibited higher oxidative potential compared to larger PM fractions of the same material [79]. Recent attention has been given to proinflammatory and endocrine-disrupting properties of diesel emissions, a major source of UFPs in ambient air [31, 99–101].

Polycyclic aromatic hydrocarbons (PAHs) are organic substances that constitute a class of over 100 individual chemical compounds made up of carbon and hydrogen atoms formed into rings [102]. While toxicological data exist for individual PAHs (benzo[a]pyrene being the most commonly used PAH indicator), they almost always occur as complex mixtures (e.g., soot, tobacco smoke, creosote, and diesel exhaust) [103]. Thus it is difficult and arguably futile to assess the toxicity of individual PAH components only to be compounded by the likelihood of interactions [75, 104, 105]. Combustion of organic matter and fossil fuels is the main source of atmospheric PAHs with their distribution and magnitude concentrated along transportation corridors (road and rail) and land-use areas with heavy industrial activities. However, main stream and environmental tobacco smoke (ETS) remain a leading source of PAH exposure [106]. PAHs are generally nonvolatile (i.e., stable) and have low water solubility. As a consequence, PAHs often bind to PM_2.5_ and UFP in the atmosphere. Residency times in the atmosphere depend on weather conditions, PAH molecular weight, and the emission source (e.g., stack versus tailpipe) with atmospheric deposition as the main source of PAHs to soil, vegetation, and surface water. Once in aquatic systems, PAHs are often found absorbed to suspended particles or bound to sediments settled on the bottom where they persist or are slowly biodegraded by microorganisms. While PAHs can bioaccumulate in some aquatic and terrestrial organisms, they tend to not biomagnify in food systems due to their metabolism in higher order species [102, 106]. However, it is the inefficient clearance and action of the highly reactive PAH metabolites that are suspected to cause cytotoxicity, mutagenicity, DNA damage, oxidative stress, and tumorgenesis [75, 106].

Much of the work elucidating the mechanisms in which PM and PAHs elicit adverse cellular effects have been conducted using cardiovascular disease (CVD) and lung cancer as models [76–78, 97, 107–109]. Although seemingly different diseases, they share several similarities with DDP and APOs with respect to associated risk factors. First, both APOs and CVD related outcomes are associated with PM exposure levels which vary by SES [40, 110, 111] but are also associated with other socially patterned risk factors such as smoking, poor or inadequate diet, psychosocial stress, obesity, and diabetes [12, 112–114]. CVD and APOs also share many other risk factors such as the presence of systemic inflammation and preexisting hypertension. Interestingly, PIH/PE is a risk factor for maternal CVD later in life and also in the offspring if affected by IUGR [115–117]. CVD and disorders of DDP have similarly affected cellular tissues in their respective target systems (i.e., endothelial cells of the cardiovascular system and in the highly vascularised placenta) which are particularly susceptible to oxidative and inflammatory injury [97, 118]. High plasma homocysteine concentrations are positively associated with vasculopathy and infarction in the placental-uterine and coronary systems increasing the risk of spontaneous PTB and CVD events, respectively [119, 120]. Fittingly, high density lipoprotein cholesterol may be protective against spontaneous PTB and CVD events [120, 121]. Finally, PM and PAH-induced mutagenicity, cytotoxicity, DNA damage, and oxidative stress linked to lung cancer have also been observed in the fetal-placental unit [122, 123], and exposure early in pregnancy may contribute to the risk of congenital anomalies and early (subclinical) pregnancy loss [124–127].

### 2.3. The Social Environment: Socioeconomic Status, Diet, Smoking, and Allostatic Load

The social environment plays a significant role in maternal and perinatal health with indicators of low socioeconomic status (SES) consistently among the strongest predictors of adverse pregnancy outcomes [10–12]. The causal pathways in which SES contribute to APOs and ill health in general can be conceptualized in terms of “downstream” or mediating exposures, stresses, and behaviours acting on the individual through “upstream” society-level determinants such as poverty, poor education, income inequality, and social discrimination/marginalization over the lifespan [12]. Indicators of low SES associated with PTB and FGR include maternal anthropometry (prepregnancy BMI, height, and gestational weight gain), nutrition and micronutrient status, cigarette use, genital tract infections and inflammation, cocaine and other drug use, physically demanding work, quantity and quality of prenatal care, and psychosocial factors including anxiety, depression, and stress (e.g., lack of social, familial, and marital support, poverty or financial hardship, physical/verbal abuse, and neighbourhood crime) [12, 24, 26, 54]. For the purpose of this review, the focus here will be on three that engage with the oxidative stress and inflammation pathways to potentially interact with exposure to particulate air pollution. They include (1) a diet-micronutrient pathway [55, 128–131], (2) cigarette smoke exposure [35, 132–135], and (3) stress-mediated (allostatic) activation of the HPA-axis and corresponding glucocorticoid production [47, 72, 136–138].

Nutrition and diet can influence perinatal health in opposing directions. Poor/under-nutrition such as high fat/calorie dense food and low micronutrient intake is more prevalent among women from low SES backgrounds which may partly explain higher rates of some APOs [12, 139–142]. Conversely, adequate diet and micronutrient status provides resilience against oxidative stress and inflammation caused by various exposures including air pollution, allostatic stress, infection, or smoking [55, 118, 128, 129, 131, 143]. Maternal exposure to mainstream or environmental cigarette smoke during pregnancy is associated with numerous APOs including congenital anomalies [127, 144–146]. Their exposure prevalence is associated with indicators of low SES as well as other socially patterned risk factors [147–149] and remains one of the most modifiable risk factors with potential for beneficial intervention. Other risk factors associated with low SES such as obesity, pre-existing and gestational diabetes, and hypertension [13, 113, 150] also engage the oxidative stress and inflammatory pathways and could therefore also potentially interact with PM exposure to increase susceptibility to adverse effects as evidenced in studies of cardiovascular health [114, 151, 152]. Recent studies have observed increased risks of preeclampsia and gestational diabetes associated with measures of air pollution [153–156] with one study showing positive effect modification by preexisting and gestational diabetes [154]. Evidence shows that chronic life stressors associated with low SES at multiple levels of organization (individual, household, and community) result in a cumulative biological toll on the body affecting multiple systems and increasing susceptibility to numerous ailments [21, 157–160] including APOs [15, 26, 161, 162].

The concept of allostasis and allostatic load/overload has been proposed to describe the individual stress response to an event as a necessary and adaptive process thereby removing the implicit negative connotation attached to the term “stress” [163]. Stress can be positive or tolerable when it improves function and performance and may have long-term adaptive benefits. However, this may depend on available coping resources such as one's psychological resistance, resilience, and ability to recover. Negative or toxic stress occurs when real or perceived environmental/social demands, or the anticipation of such, become too extreme or unpredictable thereby exceeding one's (perceived) ability to cope (e.g., no sense of control, adverse childhood experiences, and other forms of trauma) [164, 165]. Therefore, allostasis is the multisystem biological response that promotes adaptation using system mediators such as cortisol, (nor)epinephrine, vasopressin, renin, and glucagon, whereas allostatic load and overload is the cumulative toll (wear and tear) on biological systems after prolonged or poorly regulated (hyper/hypoactivated) allostatic responses [165, 166]. For example, the cardiovascular system is extremely sensitive to stress in terms of increased blood pressure; however, metabolic disorders such as diabetes and obesity as well as immune function impairment are also linked to chronic stress. Furthermore, lifestyle coping mechanisms as a response to chronic stress have the ability to either buffer or exasperate the effect (e.g., exercise, diet, sleep, and social interactions or lack thereof) [163]. Therefore in light of the above, it is our belief that the fetal-placental unit is the site where the physical and social environments converge and interact to influence reproductive health which we describe further below.

**Figure 1:**
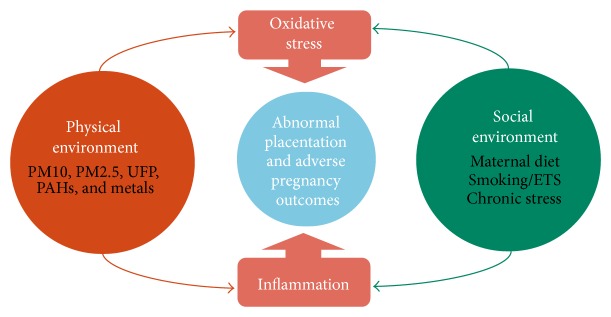
A conceptual framework of the shared mechanisms of socioeconomic determinants and particulate air pollution exposure contributing to adverse pregnancy outcomes. The physical environment (orange) consisting of particulate air pollution and the social environment (green) consisting of community and individual-level social factors/stressors converge to affect the fetal-placental environment (blue) via oxidative stress and inflammatory mechanisms potentially leading to adverse pregnancy outcomes.

Figure 1 illustrates the interconnectedness between particulate air pollution (PM/PAH) and SES on how they may act discretely or in a combined manner to yield APOs. Using Figure 1 as a guide, the following text will review the two major mechanisms (oxidative stress and inflammation) through which the physical and social environments are believed to negatively affect the fetal-placental unit and how they may combine/interact to lead to the multifactorial nature of APOs.

## 3. Biological Mechanisms Leading to Adverse Pregnancy Outcomes

### 3.1. Oxidative Stress

Aptly known as “The Oxygen Paradox,” oxygen is both essential and toxic to the multicellular aerobic organisms whose very evolution was dependent on leveraging this anaerobic waste by-product into a higher energy producing advantage [167]. Observed in all mammals, a steep oxygen tension gradient from 20% in our atmosphere to 3-4% oxygen concentration in most internal tissues is the primary defense against oxidative damage. Secondary and tertiary layers of protection include antioxidant defenses as well as damage removal, repair, and apoptotic response systems [168, 169]. These genetically adaptive responses are upregulated in the presence of reactive oxygen species (ROS) generated as natural by-products of cellular aerobic metabolism and exposure to various toxins. Oxidative stress occurs when there is an imbalance between pro- and antioxidant capacity.

For example, superoxide is the most common intracellular ROS in mammals. It is produced by the mitochondria as a metabolic by-product but also from the metabolism of various growth factors, drugs, and toxins by oxidizing enzymes such as NADPH-oxidase and cytochrome P450 (CYP450). Superoxide is reduced by superoxide dismutase (SOD) into hydrogen peroxide (H_2_O_2_) which is then further reduced into water by glutathione peroxidase (GPx) and catalase. Under normal physiological conditions H_2_O_2_ acts as intracellular secondary messengers; however, it's accumulation along with superoxide can react with free iron ions or nitric oxide to form highly toxic hydroxyl (OH^∙^) or peroxynitrite (ONOO^−^) ions, respectively [70, 168]. Free iron is a common metal found absorbed to PM, and the antioxidant heme oxygenase-1 (HO-1) facilitates its conjugation and removal through the increased availability of ferritin thereby preventing the formation of reactive hydroxyl molecules [92, 170–172]. Deficiencies in HO-1 have been associated with several APOs such as recurrent miscarriage, FGR, and preeclampsia [171, 172].

Common antioxidants include enzymatic (e.g., SOD, GPx, catalase, and HO-1) and nonenzymatic compounds (e.g., vitamin C and E, glutathione, *β*-carotene, and ubiquinone) [118]. Genetic polymorphisms and/or micronutrient deficiencies in antioxidant enzymes precursors can impair antioxidant capacity, while chronic exposure to toxicants, psychosocial stress, bacteria, viruses, and other inducers of inflammation can foster prooxidant burden [70, 77, 118, 172]. Oxidative stress is unavoidable; however, under optimal conditions the presence of ROS leads to homeostatic adaptation and are safely removed. Failure to effectively manage oxidative stress can result in altered cellular function as excess ROS degrade lipids, proteins, and DNA potentially initiating pathological processes. Refer to [168] for an extensive review on the role of cellular ROS in pregnancy outcomes.

### 3.2. Inflammation and Immunologic Alterations

It is well recognized that the maternal immune system plays a central role throughout the entire pregnancy, from preimplantation to parturition, and is influenced by the inflammatory response of the mother to her environment as well as to her partner. Alternative to previously hypothesized [173], the maternal immune system is not passive or suppressed during implantation and development of the semiallogeneic placenta and fetus. Rather, it exerts executive influence on the establishment and progression of the pregnancy as an immune-mediated quality control mechanism to maximize maternal and offspring health [44, 173]. This is achieved by favouring pro- or anti-inflammatory environments at different times during pregnancy for different purposes. For instance, implantation, initial placentation, and parturition are characterized by a proinflammatory environment whereas an anti-inflammatory state prevails for most of midgestation [174]. The favoured localized immunological response however is highly modified by the infectious, inflammatory, stress, nutritional, and metabolic status of the individual and thus can be influenced by environmental agents such as PM [175–177] and/or available coping, social, and nutritional resources [44, 128, 164, 178]. Therefore, inflammation is believed to be one pathway involved in both PM and SES-mediated APOs.

**Figure 2:**
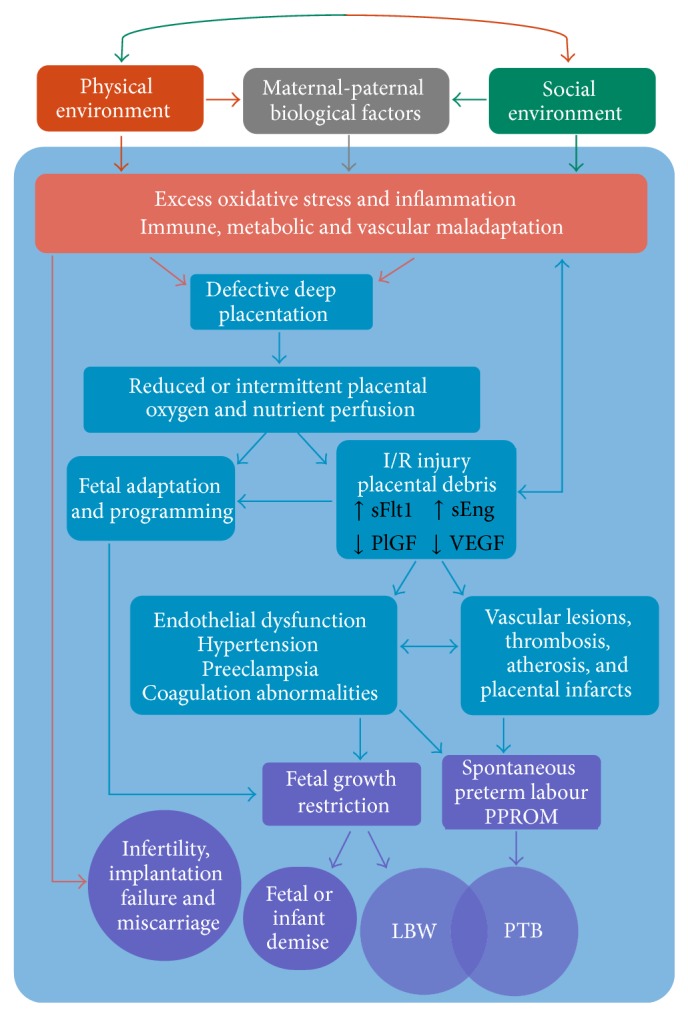
Proposed pathways contributing to adverse pregnancy outcomes. The co-presence of maternal and paternal biological factors can result in protection or increased susceptibility to the interaction with the physical and social environments. Cumulative negative exposures early in pregnancy resulting in excess oxidative stress and inflammation may cause a cascade of events leading to defective deep placentation. Depending on the degree of severity, the reduced transplacental perfusion can result in various pathologies associated with a range of obstetric complications and outcomes [60, 61, 69, 70].

Chronic and acute inflammation is a complex response process mediated by a real or perceived attack from foreign substances. The innate immune response is the rapid automatic response to externally originating (exogenous) substances such as pathogens, but also from internal (endogenous) danger signals including products of trauma, ischemia, necrosis, or oxidative stress [179]. The response includes the release of proinflammatory signaling cytokine proteins such as interleukins IL-1*β*, IL-6, and tumour necrosis factor (TNF-*α*) which serve to recruit neutrophils to affected tissues. However, the recruited neutrophils release ROS and hydrolytic proinflammatory enzymes (inducible nitric oxide synthase (iNOS), cyclooxygenase (COX-2), and prostaglandins (PG-E_2_)) which disturb normal cells in addition to affected tissues. This in turn leads to increased ROS and oxidative stress [180, 181]. The placenta plays a role at the maternal-fetal interface as the main producer of endocrine steroid and protein hormones as well as the immunologic barrier between mother and fetus which positively interact for the success of the pregnancy [44, 173]. This is achieved through a nonlinear series of positive and negative feedback pathways with the stimulation or suppression of molecules with pro- and anti-immunosuppressant properties (interleukins, galectins, placental growth factor, and human chorionic gonadotropin (hCG)) [182–184]. The production of these cytokines, chemokines, and other immune-regulatory agents mediates the coordination, migration, and function of several maternal immune cells (e.g., uterine natural killer cells (uNK)) that participate in early pregnancy events such as endometrial receptivity of embryo implantation, tissue remodeling, immune tolerance, and vascular adaptation to invading placental trophoblast cells [44, 182–184]. Interference or aberrant production/secretion of these substances by various stressors including infection, toxins, and those acting through the HPA-axis may result in the impaired maternal immune response leading to the hallmark DDP syndrome complications described above (early pregnancy loss, PIH/PE, PROM, FGR, and premature labour, Figure 2) [44, 61, 69, 134, 175, 185–187].

### 3.3. Mechanisms of Oxidative Stress and Inflammation Involved in Adverse Perinatal Outcomes

#### 3.3.1. Impaired Fertility and (Recurrent) Miscarriage

Due to immortal time bias, miscarriage is not easily measured in population or cohort studies without careful design methodologies [188, 189]; however, associations between infertility and air pollution have been made [190, 191]. Oxidative stress has shown to have a direct effect on fertility and embryo development. For example, obese mice showed increased ROS synthesis and oxidation in oocytes with a reduced ability of zygotes to develop to the blastocyst stage providing evidence that impaired cellular antioxidant capacity can limit successful ovulation and fertilization [118]. Dividing mitotic cells are particularly sensitive to oxidative damage and are shown to enter a transient growth-arrested state as a protective mechanism until the stress has passed. Thus, severe or chronic oxidative stress may hamper cell division or cause cellular necrosis reducing or terminating embryo viability [72, 169]. Alternatively, an exaggerated inflammatory state via a viral, toxic, and/or allostatic load could lead to a maternal immune maladaptation to conception restricting trophoblast stem cell accumulation in the early embryo responsible for the production of hormones that enables successful implantation (Figure 2) [44, 72].

Oxidative stress is implicated in first trimester miscarriage from premature placental perfusion of maternal oxygenated blood and accompanying ROS into the early embryonic environment [192]. Early embryo development occurs in a low oxygen state, and it is not until the tenth to twelfth week of gestation that maternal blood begins to gradually infiltrate the intervillous space of the yet fully developed placenta. The limited oxygen environment is thought to act as a protective mechanism against the deleterious and teratogenic effects of ROS on early stem cells at a time of extensive cell division [64, 138]. This early hypoxic environment also plays a vital physiological role in placental cell type differentiation switching from proliferative villous cytotrophoblasts into invasive extravillous trophoblasts (EVT) important in spiral artery remodeling [193]. At the end of the first trimester, oxygen tension rises sharply which coincides with the infusion of oxygenated maternal blood into the placenta and triggers an apoptotic cascade that serves to establish the definitive discoid placenta. However, in 70% of early miscarriage cases EVT invasion is insufficient allowing for the premature onset of maternal intraplacental circulation and its consequential burst of ROS on the conceptus [70, 192]. Severe cases may result in pregnancy failure while more modest cases may initiate fetal-maternal adaption to impaired spiral artery remodeling leading to the DDP pathology and further complications later in pregnancy such as FGR and PIH/PE (Figure 2) [69, 70, 193].

#### 3.3.2. Pregnancy Induced Hypertension, Preeclampsia, and Prelabour Rupture of Membranes

While oxidative stress and inflammation are conditions of normal pregnancy, they are consistently elevated in cases of PIH/PE and central in its pathology. PIH/PE stems from a defect in early trophoblast invasion insufficient to fully convert the spiral arteries into low-resistance channels [68, 194]. The retention of smooth muscle cells remains active to circulating vasoconstricting agents such as stress hormones (e.g., glucocorticoids) and other stimulants. The diminished and intermittent perfusion of maternal blood into the intravillous space produces transient hypoxia resulting in a chronic ischaemia-reperfusion (I/R) type injury. This further provokes ROS synthesis and excess shedding of placental microvesicles which have proinflammatory, antiangiogenic, and procoagulant activity initiating endothelial dysfunction [68–70]. Elevated circulating levels of placental debris and ROS biomarkers in the placental tissues of preeclamptic women are well documented [68, 179, 194]. Similarly, PROM can be considered part of the DDP syndrome but may represent a phenotype resulting from a less severe DDP pathophysiology compared to preeclampsia [61, 62]. Excess oxidative stress arising from multiple causes (infection, inflammation, smoking, and cocaine use) has been implicated in PROM in addition to its role in DDP [70]. Both PIH/PE and PROM are leading causes of preterm birth, while PIH/PE is a major risk factor for FGR (Figure 2) [69]. Deficiencies in HO-1 have been associated with preeclampsia as well as morphological changes in the placenta and elevations in maternal blood pressure. The bioactive HO-1 metabolites CO and bilirubin may protect against preeclampsia through their vasodilatory properties and the suppression of the antiangiogenic factor sFlt, respectively [171, 172].

#### 3.3.3. Fetal Growth Restriction

FGR has many causes however often arises from placental insufficiency due to compromised supply of oxygen and nutrients to the fetus which may have both short- and long-term health consequences on the offspring [51, 82, 195]. FGR is strongly associated with early onset or more severe cases of preeclampsia, and there is a clear etiological link between FGR and DDP as it involves abnormal placentation and reduced uteroplacental blood flow (Figure 2) [62, 70]. Alternatively, perturbed calcium homeostasis can induce chronic low-level stress within the endoplasmic reticulum leading to suppressed protein synthesis and a reduced growth trajectory of the placenta [70]. Cadmium, an environmental toxin and highly present in cigarette smoke, is a major antagonist of cellular calcium activities (transport, uptake, and binding) as well as in the transfer of other nutrients and zinc homeostasis within the placenta [134, 185, 196]. Furthermore, cadmium is a known endocrine disruptor shown to impair hormone synthesis in the placenta including progesterone and leptin, important hormones in early pregnancy [49, 175, 186]. Both smoking and air pollution exposure were associated with lower birth weights along with low blood progesterone levels and high placental cadmium concentrations compared to a non-exposed control group [135].

#### 3.3.4. Spontaneous Preterm Labour and Birth

Inflammation is proposed as one potential mechanism leading to spontaneous preterm labour, both with intact membranes or PROM [177]. The classification of patients who deliver preterm can be categorized into two non-mutually exclusive clusters: those who present with inflammatory lesions (e.g., acute chorioamnionitis and funisitis) and those with vascular lesions who tend to have longer gestational periods [61]. The consequence of uteroplacental ischemia as a result of such lesions will depend on the severity, the timing, and duration of the insult. While a complete blockage of uterine arteries will lead to fetal death, less severe ischemia will result in different clinical phenotypes as a result of adaptive mechanisms for fetal survival. This may include fetal growth restriction if chronic underperfusion of oxygen and nutrients persists, the onset of maternal hypertension to sustain or increase uterine blood flow, and/or the initiation of preterm labour as a maternal/fetal adaptation to continued growth restriction* in utero *(Figure 2) [61, 197]. Cardiovascular lesions indicating thrombosis and atherosis are shown to be indirectly caused by exposure to PM_2.5_ and UFPs via inflammatory and/or oxidative injury [97].

## 4. The Physical and Social Environments and Their Relation to Adverse Perinatal Outcomes

### 4.1. PM-Induced Oxidative Stress and Inflammatory Mechanisms

**Figure 3:**
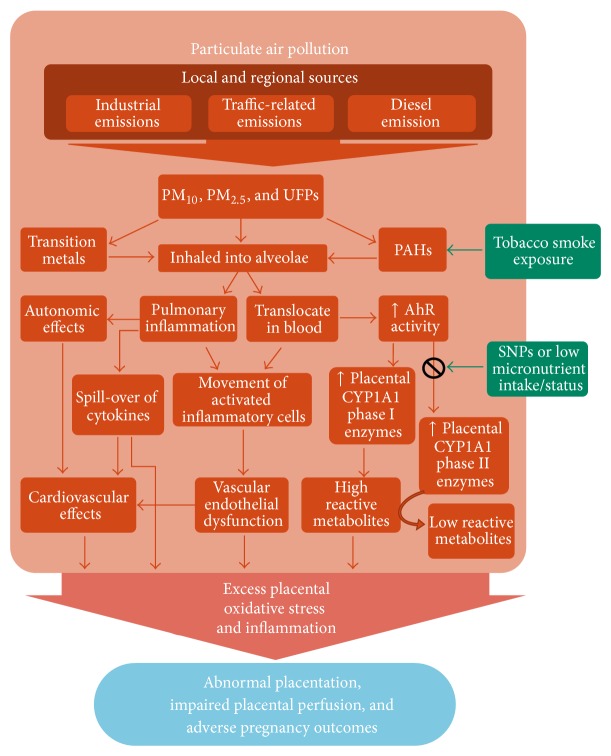
Proposed pathways of particulate air pollution contributing to oxidative stress and inflammation leading to adverse pregnancy outcomes. Exposure to PM and its associated constituents of transition metals, PAHs, and other organic molecules affect the cardiovascular and metabolic systems which are highly active throughout pregnancy. For example, detoxification of PAHs and other organic toxins activate AhR signalling resulting in additional oxidative stress if antioxidant defenses are limited or impaired [55, 79, 98, 108, 109, 199].

Exposure to PM_2.5_ and its constituents, including PAHs and metals, induce oxidative stress and inflammation in many biological systems through various means (Figure 3) [48, 77–79, 97, 176, 177, 198]. One method is the direct generation of ROS from free radicals and oxidants on particle surfaces including soluble transition metals such as iron, copper, chromium, and vanadium. As mentioned above, free iron can react with available superoxide or hydrogen peroxide to form highly reactive hydroxyl radicals [70, 77]. PAHs and other organic molecules absorbed to PM_2.5_ and UFPs may account for a large proportion of their oxidative potential due to their ability to enter the cell and disrupt the mitochondria [79]. Altered function of mitochondria may produce excess quantities of NADPH-oxidase which in turn generates large amounts of cellular superoxide, a process already in overdrive throughout pregnancy but particularly in the first trimester [70, 77]. Interpolated ambient PM_10_ exposure was shown to be negatively associated with the number of placental mitochondrial DNA, a molecular marker of mitochondrial disruption and inflammation. This association was reversed with increasing distance from major roads, a proxy for traffic-related air pollution [48].

Alternatively, PM/PAH mediated oxidative stress can be induced by the activation of the inflammation system. Immunotoxic compounds can promote the release of proinflammatory cytokines, TNF-*α*, and COX-2, which in turn act in a positive feedback loop to generate more ROS and oxidative stress [77]. For example, modelled PM_10_ and PM_2.5_ exposure has been positively associated with elevated C-reactive protein (CRP) levels, a biomarker of systemic inflammation, in both maternal first trimester blood and fetal cord blood in a dose-dependent manner [176, 200]. CRP is produced in the liver and part of the acute-phase response released during inflammatory reactions from cytokines produced in the lungs. Raised CRP is a risk factor for cardiovascular disease as a marker of unstable atheromatous plagues leading to thrombosis and ischemic events [97]. Exposure to diesel exhaust in healthy human volunteers resulted in pulmonary inflammation in addition to systemic inflammation, prothrombotic changes, and other cardiovascular effects consequent of proinflammatory events [99, 201]. This hyper proinflammatory state, along with oxidative stress, is hypothesized to contribute to several APOs [69, 70, 174, 181, 202].

Indirectly, the cellular detoxification of PAHs can induce oxidative stress and cytotoxicity by forming potent ROS metabolite by-products. Specifically, PAHs and other organic xenobiotics (notably PCBs and dioxins) are detoxified by the cytochrome P-450 (CYP) superfamily of Phase I and Phase II metabolizing enzymes. The expression of these enzymes is highly modulated by genetic polymorphisms, steroid/sex hormones such as glucocorticoids, insulin, estrogens, and progesterone, and micronutrient/dietary deficiencies [74, 75, 128, 203, 204]. Furthermore, hypoxia, infection, and inflammation are shown, in general, to downregulate CYP enzymes which may affect the clearance and bioavailability of growth factors, hormones, drugs, and toxins [203, 205]. CYP has numerous isoforms which are expressed in many tissues especially the liver. CYP1A1 is the only isoform also significantly expressed in the placenta throughout pregnancy responsible for metabolizing steroid/sex hormones, growth factors, and fatty acids in addition to toxins [75]. These exogenous and endogenous substances act as ligands to activate the aryl hydrocarbon receptor (AhR), a transcription factor that mediates the biotransformation of such ligands (PAHs, estradiol, etc.) into more polar and bioavailable metabolites by upregulating CYP enzymes. However, certain metabolites of PAHs (e.g., o-quinones, arene oxide, and diol epoxide) bind to DNA, RNA, and protein macromolecules to form toxic adducts that disrupt DNA replication and are considered mutagenic [72, 75]. Such DNA adducts have been found in newborn cord-blood positively correlated with maternal exposure to PAHs [50]. PAHs have also shown to significantly decrease the accumulation of trophoblast stem cells in the early placenta thereby limiting their differentiation into other cell types vital for hormone synthesis and ongoing placental development, a process that could contribute to DDP [72]. Direct prenatal exposure to airborne PAHs has been associated with FGR with an increased exposure-related risk in the first trimester [206, 207]. Secondary (Phase II) metabolizing enzymes are required to further detoxify reactive PAH-metabolites in which their inefficient clearance results in prolonged exposure leading to sustained cytotoxicity and mutagenicity. Phase II enzymes include glutathione s-transferases (GSTs), UDP-glucuronosyltransferases (UGTs), NAD(P)H-dependent quinone oxidoreductase-1 (NQO1), and aldehyde dehydrogenase-3 (ALDH3) [75, 205].

### 4.2. Maternal Diet and Micronutrient Intake

**Figure 4:**
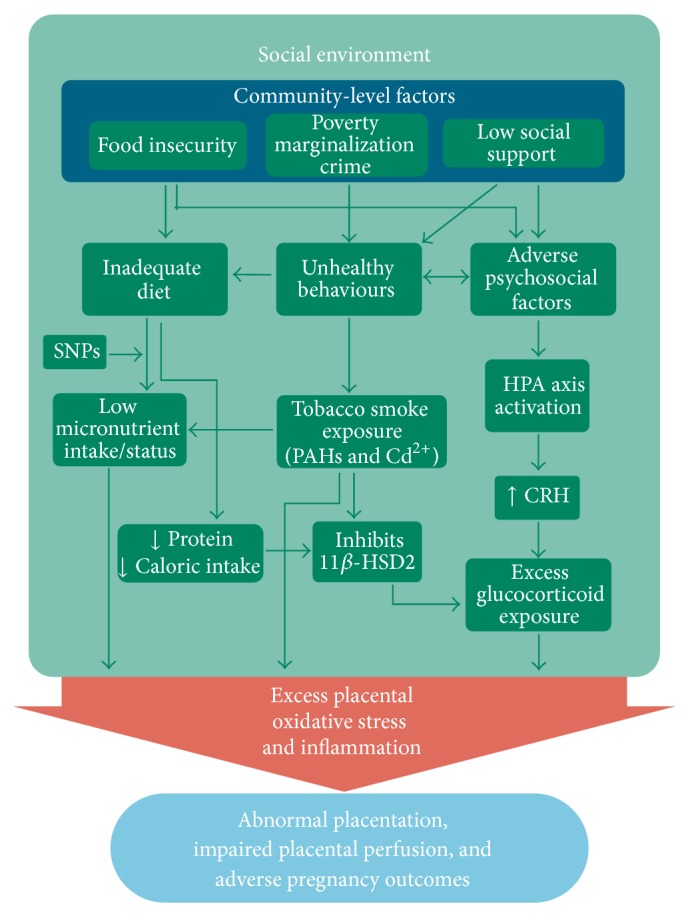
Proposed pathways of how the social environment interacts to produce excess systemic and placental oxidative stress and inflammation leading to adverse pregnancy outcomes. The pregnant woman is nested within and influenced by neighbourhood/community-level factors which can exasperate or buffer the individual-level biological and behavioural factors [24, 26, 54, 128, 224, 225].

Adequate diet and micronutrient status provides resilience against oxidative stress and inflammation caused by various exposures including air pollution, allostatic stress, infection, and smoking (Figure 4) [55, 118, 128, 129, 131, 143]. Many micronutrients such as essential trace metals are vital cofactors in several antioxidant enzyme systems. For example, copper and zinc are necessary in the production of SOD. Similarly, selenium and its incorporation into the amino acid selenocysteine are required for the functionality of all selenoenzymes, including GPx and GST. Thus, selenium is essential in several aspects of human health, particularly conditions involving oxidative stress and inflammation such as CVD, immune function, cancer, and reproduction, but also thyroid regulation and brain diseases [208, 209].

ROS may have direct effects on oocyte quality and appears to be modulated by dietary antioxidant supplements [118]. Women who are obese tend to have higher rates of infertility that correlate with increased levels of oxidative stress biomarkers in their blood as excess glucose availability leads to higher mitochondrial ROS synthesis [70, 118]. Selenium deficiency and corresponding reduced GPx activity has been documented in cases of recurrent miscarriage and spontaneous abortions [210–212] and has also been associated with preeclampsia and preterm birth [213, 214]. However, given the supposed role of oxidative stress in preeclampsia, treatment with certain antioxidants (notably vitamins C and E) has not produced reliable preventative results in experimental trials [69]. One hypothesis is that inappropriate antioxidant regiment and/or administration too late in gestation are responsible and new therapeutic candidates include melatonin and selenium [118]. Interestingly, national programs in Finland and New Zealand fortifying food with selenium have been associated with a significant reduction in the rate of preeclampsia [215].

Oxidative stress negatively affects the placental transport of amino acids and glucose [45]. Furthermore, fatty acids and low density lipid (LDL) cholesterols necessary for the placental synthesis of oestrogens and progesterone are particularly vulnerable to oxidative injury [216]. Regulation of placental nutrient transport is controlled by several different mechanism, including imprinted genes, placental signaling pathways, various cytokines, and hormones such as insulin, leptin, glucocorticoids, and oestrogens (for review see [45]). The major placental transfer mechanisms include simple diffusion of lipophilic substances (e.g., oxygen, CO_2_, fatty acids, steroids, fat soluble vitamins, and anesthetic gases), restricted diffusion of hydrophilic substances, facilitated diffusion via a membrane bound carrier (e.g., glucose and other carbohydrates), and active transport which requires energy (e.g., amino acids, iron, calcium, and other divalent cations) [45, 217]. Placental physiology, including spiral artery remodeling and placental villous surface area are major determinants dictating placental transport capacity, and the degree of placental developmental disruption correlates with the severity of obstetrical complications associated with DDP [51, 62].

Nutrition and diet can influence perinatal health in opposing directions (i.e., it can be an antagonist or agonist). Poor/undernutrition such as high fat/calorie dense food and low micronutrient intake is more prevalent among women from low SES backgrounds which may partly explain higher rates of some APOs [12, 139–142]. On the other hand, good nutrition and supplemental vitamin intake is capable of reducing the toxicity of everyday environmental stressors as well as preventing certain APOs and congenital anomalies as shown with the successful reduction of neural tube defects with folic acid [128, 143, 218]. Nutritional and/or genetically induced deficiencies in folate and vitamins B6 and B12 can disrupt the homocysteine-to-methionine pathway resulting in hyperhomocysteinemia (HHC), a known risk factor of cardiovascular morbidities (thrombosis, lesions, and infarcts) and markers of oxidative stress [54, 119, 219, 220]. HHC may similarly affect the highly vascularized placenta and has been associated with decidual vasculopathy and preterm birth [54, 120]. Omega-3 fatty acids abundant from eating salmon were shown to improve markers of oxidative stress [221], which may impart neurodevelopmental resilience against stressors [222, 223]. Dietary phytophenols from fruits, vegetables, herbs, and spices have shown to have antioxidant and anti-inflammatory properties capable of reducing infection-induced inflammatory and contractile pathways in human gestational tissues [129]. Significant differences in pregnancy outcomes between Dominicans and African Americans both exposed to similar levels of PAHs in New York city neighbourhoods were thought to be due to healthful dietary/cultural practices in the Dominican immigrant population [206].

### 4.3. Maternal Smoking and Environmental Tobacco Smoke (ETS) Exposure

Maternal smoking during pregnancy and exposure to ETS remain to be two modifiable risk factors with the greatest potential for beneficial interventions (Figure 4). Their association with numerous APOs including congenital anomalies is well documented [127, 144–146], as have their associated prevalence with indicators of low SES and other socially patterned risk factors [147–149]. The mechanisms involved leading to APOs have been well reviewed [132, 134]; however, it is notable that the two main toxins present in tobacco smoke can also be absorbed to PM_2.5_ (PAHs more so than cadmium). Cadmium (Cd) exposure readily interferes with the active transport of essential minerals to the fetus, particularly zinc and calcium [46, 135, 196, 226–228]. Cadmium and lead (Pb) exposure has also been shown to reduce glycogen concentrations thereby potentially limiting available glucose to the fetus [229]. Cadmium has shown to disrupt placental leptin synthesis, a hormone with several vital functions including placental angiogenesis, immunomodulation, amino acid and fatty acid transport, as well as fetal pancreatic development important in the regulation of insulin-like growth factors and fetal body fat accumulation [49, 51]. Finally, synergistic effects in the generation of oxidative hydroxyl radicals have been observed between tobacco smoke and both ambient PM_2.5_ and diesel exhaust particles specifically [35]. Interestingly, the counterintuitive association between smoking and lower risk of preeclampsia was recently shown to vary according to the timing and intensity of smoking [230]. It is possible that the increased exposure to CO from smoking in late gestation acts as a vasodilator and at the same time inhibits the release of sFlt-1, a hallmark antiangiogenic factor implicated in the endothelial dysfunction present in preeclampsia [115, 230].

### 4.4. Allostatic Stress and Glucocorticoid Exposure

Reviewed elsewhere [163], the brain is the primary target and mediating organ through which SES-related stress pathways are translated to other body systems via the hypothalamic-pituitary-adrenal (HPA) axis. The HPA-axis is actively involved in several biological systems, including the cardiovascular, metabolic, immunological, and endocrinal effects in both mother and fetus to promote allostatic adaptation [165, 231]. Here, the neuroendocrine hormones of the HPA axis, corticotrophin releasing hormone (CRH), adrenocorticotropic hormone (ACTH), and glucocorticoids (GC), respectively, coordinate the biological response via feedback loops. The human placenta is also capable of releasing CRH and other neuropeptides which interact with the HPA axis to regulate the maternal stress response as well as other normal pregnancy functions [47]. Proper levels of* in utero* glucocorticoids are essential for successful embryo implantation, fetal organ maturation, and the initiation of labour with glucocorticoid levels gradually increasing over the course of gestation. Normally, levels of maternal cortisol rise sharply in the third trimester causing the release of placental CRH in a positive adrenal-placental feedback loop. Placental CRH stimulates fetal cortisol secretion which in turn suppresses placental progesterone and activates the release of prostaglandins and oxytocin to promote uterine contractions [47, 232]. However, early and increased levels of fetal glucocorticoids can impair growth and predispose to adult-onset diseases [136, 233, 234]. The placental enzyme 11*β*-hydroxysteroid dehydrogenase type 2 (11*β*-HSD2) protects the fetus from excess endogenous glucocorticoids by converting active cortisol into inactive cortisone. 11*β*-HSD2 is hormonally regulated making it susceptible to endocrine disruption from chemical and nonchemical stressors such as maternal anxiety, inflammation, infection, cadmium exposure, and low caloric intake [136, 138, 224, 235, 236]. Placental hypoxia associated with PIH/PE has been shown to suppress 11*β*-HSD2 activity which may be an adaptive response to counteract compromised fetal growth by allowing more cortisol to reach the fetus for organ development. Low concentrations/activities of 11*β*-HSD2 and high levels of cortisol have been associated with PTB and FGR [136, 237, 238], two outcomes also associated with poor maternal psychosocial/mental health [233, 234, 239].

Factors affecting 11*β*-HSD2 activity that are associated with low SES include allostatic overload leading to the excess production of glucocorticoids that can overwhelm the fetal protective mechanism (Figure 4) [136, 231, 240]. Indirectly, allostatic load is capable of disrupting the metabolic system leading to impaired glucose tolerance, insulin resistance, diabetes, and/or obesity, all of which are risk factors for various APOs [138, 165, 231]. General maternal undernutrition and/or a low dietary protein intake has been shown to impair placental glucose transport and inhibit 11*β*-HSD2 activity in pregnant rats leading to FGR, indicating a possible mechanism through poor diet [224, 241]. Additionally, cadmium has also shown to inhibit 11*β*-HSD2 activity in both human and rodent placentas [225], and prenatal cadmium exposure has been shown to increase fetal corticosterone concentrations in rats which resulted in reduced birth weights [236]. This suggests a possible mechanism from active or passive tobacco smoke exposure or ambient PM_2.5_ exposure [135, 242, 243]. Collectively, it is possible for the cumulative exposures of PM_2.5_, smoking, ETS, poor dietary intake, and other SES-related factors to interact through the same 11*β*-HSD2 mechanism to increase the risk of impaired fetal growth (Figure 4).

## 5. Discussion

The ubiquitous exposure to particulate air pollution and its constituents (e.g., PAHs and metals) is but one class of environmental contaminants that can act through oxidative stress, inflammation, and/or endocrine disruption to promote developmental toxicity and adverse perinatal health [177, 244, 245]. Summarized in Figure 2, a perturbed early* in utero* environment can lead to defective deep placentation resulting in a cascade of fetal-placental adaptive mechanisms contributing to a range of pregnancy complications and adverse outcomes [60]. Here the underlying biological, social, and physical risk factors likely intersect to produce excessive or atypical oxidative stress, inflammatory response, and biological antagonism to either initiate the defective deep placentation pathology and/or contribute to the severity of its phenotype. Socioeconomic disparities are known to confound the environmental exposure effects; however, they may also act as potential effect modifiers given their overlapping etiological mechanisms with PM_2.5_ exposure. While the traditional biomedical paradigm that views populations as a collection of independent individuals has yielded useful information regarding risk factors, elucidating the intersecting pathways involved in APOs will require placing individual biologic and behavioural determinants within the social and spatial context [22, 246]. It is now well recognized that SES operates at multiple levels of organization, and neighbourhood or community-level factors can work to either ameliorate or exacerbate certain risk factors [15, 24–26]. The healthy migrant paradox exemplifies these effects in which home country, education, and neighbourhood qualities combine to modify the expected perinatal outcomes often observed with low income households [161, 247].

The SES risk factors that overlap or interact with the PM-mediated mechanisms include smoking, nutrition, and psychosocial stress acting through the HPA-axis and allostatic load. Given this knowledge, interventions aimed at ameliorating these factors may be the best way to counteract the negative influences of low SES and air pollution exposure on fetal development. Maternal smoking continues to be one of the most modifiable risk factors to lower the risk of APOs [134, 147]. Furthermore, maternal smoking also tends to interact negatively with nutrient intake and status [133, 248]. Smokers in general have poorer nutritional profiles than nonsmokers with both behavioural and biological factors independently accounting for the differences in micronutrients such as folate and essential vitamins and minerals [133, 248–250]. While smokers tend to have lower dietary nutrient intakes, they also have an accelerated requirement for micronutrients due to increased inflammatory cell turnover caused by the oxidative stress of smoking, an effect more pronounced among heavy and long-time smokers [248]. These interacting effects of smoking and nutrition are further compounded by their association with other indicators of low SES such as low education and income contributing to allostatic load [139, 251]. Nutrient intake may be ameliorative after an insult has occurred as shown in rat models of fetal alcohol syndrome where an omega-3 fatty acid enriched diet reversed the cellular effects of prenatal ethanol exposure on the fetal brain [222]. Therefore with respect to policy interventions, nutrition in the form of improved food security and micronutrient intake may serve to counteract the negative influences of both low SES and air pollution exposure [252–257].

The complex mixture of particulate air pollution, especially PM_2.5_ and UFPs which includes absorbed PAHs and various metals, also emerges as an important target for risk reduction and management. The deserved scrutiny stems from their ubiquity in the environment, the myriad of emission sources, and their established association with APOs [88, 98, 258]. The pervasiveness of PM_2.5_ and UFPs in the environment means that a high proportion of people are exposed resulting in a high etiological fraction. Therefore, even a modest reduction in exposure will have a large population effect with reduction of the societal costs of APOs [259]. Notably, their sources are primarily local, such as vehicle emissions and industrial land-use. This makes them modifiable risk factors that can be addressed at the municipal and provincial/state level with better urban planning to reduce vehicle traffic, increasing access to green-space and enforcing air quality regulations [260–263].

Not unlike the accumulation of evidence on smoking and health outcomes or that of air pollution on cardiovascular and pulmonary health [264], the epidemiological and toxicological research over the past two decades has established a consistent dose-response association with high biological specificity, temporality, and plausibility [3, 55, 177]. Taken in concert, these characteristics and further corroborating research should lend strength for evidence-based policy for intervention strategies targeting high risk areas in order to reduce the environmental burden of disease attributed to particulate air pollution [98, 265, 266].

## 6. Conclusion

Adverse pregnancy outcomes such as fetal growth restriction and preterm birth are a public health priority of global importance. We have brought together the multidisciplinary literature on the current state of evidence linking the physical and social environment to specific adverse pregnancy outcomes. The evidence suggests that various exposures, whether socially or environmentally determined, may be interpreted by the fetoplacental unit in similar ways resulting in a common pathological foundation for adverse outcomes, namely, deficient deep placentation. Given this background, well planned future epidemiology studies using multilevel models exploring various biological effects of the social and physical environment will have the potential to provide the evidence to establish crucial windows of fetal vulnerability with an aim to identify and mitigate modifiable risk factors.

## Acronyms

AhR: Aryl hydrocarbon receptor
ACTH: Adrenocorticotropic hormone
APO: Adverse pregnancy/perinatal outcome
Cd: Cadmium
CRH: Corticotropin releasing hormone
COX-2: Cyclooxygenase-2
CO: Carbon monoxide
CRP: C-reactive protein
CVD: Cardiovascular disease
CYP: Cytochrome P450
DPP: Defective deep placentation
ETS: Environmental tobacco smoke
EVT: Extravillous trophoblast
IUGR or FGR: Intrauterine or fetal growth restriction
HHC: Hyperhomocysteinemia
HPA axis: Hypothalamus-pituitary-adrenal axis
I/R injury: Ischemic-reperfusion injury
GPx: Glutathione peroxidase
GST: Glutathione-S-transferase
LBW: Low birth weight
LDL: Low Density lipoproteins
NO, NO_2_: Nitrogen oxide/dioxide
PAH: Polycyclic aromatic hydrocarbons
PAP: Particulate air pollution
PIH/PE: Pregnancy induced hypertension/preeclampsia
PM: Particulate matter
pPROM: (Premature) Prelabour rupture of membranes
PTB: Preterm birth
ROS: Reactive oxygen species
SES: Socioeconomic status
SNP: Single nucleotide polymorphism
SOD: Superoxide dismutase
sEng: Soluble endoglin
sFlt: Soluble fms-like tyrosine kinase
UFP: Ultrafine particles
uNK (cells): Uterine natural killer
11*β*-HSD2: 11*β*-Hydroxysteroid dehydrogenase type 2.
